# Sources of Variability in Metabolite Measurements from Urinary Samples

**DOI:** 10.1371/journal.pone.0095749

**Published:** 2014-05-01

**Authors:** Qian Xiao, Steven C. Moore, Simina M. Boca, Charles E. Matthews, Nathaniel Rothman, Rachael Z. Stolzenberg-Solomon, Rashmi Sinha, Amanda J. Cross, Joshua N. Sampson

**Affiliations:** 1 Nutritional Epidemiology Branch, Division of Cancer Epidemiology and Genetics, National Cancer Institute, National Institutes of Health, Rockville, Maryland, United States of America; 2 Biostatistics Branch, Division of Cancer Epidemiology and Genetics, National Cancer Institute, National Institutes of Health, Rockville, Maryland, United States of America; 3 Occupational and Environmental Epidemiology Branch, Division of Cancer Epidemiology and Genetics, National Cancer Institute, National Institutes of Health, Rockville, Maryland, United States of America; 4 Imperial College London, London, United Kingdom; Steno Diabetes Center, Denmark

## Abstract

**Background:**

The application of metabolomics in epidemiological studies would potentially allow researchers to identify biomarkers associated with exposures and diseases. However, within-individual variability of metabolite levels caused by temporal variation of metabolites, together with technical variability introduced by laboratory procedures, may reduce the study power to detect such associations. We assessed the sources of variability of metabolites from urine samples and the implications for designing epidemiologic studies.

**Methods:**

We measured 539 metabolites in urine samples from the Navy Colon Adenoma Study using liquid chromatography-mass spectrometry (LC-MS) and gas chromatography-mass spectroscopy (GC-MS). The study collected 2–3 samples per person from 17 male subjects (age 38–70) over 2–10 days. We estimated between-individual, within-individual, and technical variability and calculated expected study power with a specific focus on large case-control and nested case-control studies.

**Results:**

Overall technical reliability was high (median intraclass correlation = 0.92), and for 72% of the metabolites, the majority of total variance can be attributed to between-individual variability. Age, gender and body mass index explained only a small proportion of the total metabolite variability. For a relative risk (comparing upper and lower quartiles of “usual” levels) of 1.5, we estimated that a study with 500, 1,000, and 5,000 individuals could detect 1.0%, 4.5% and 75% of the metabolite associations.

**Conclusions:**

The use of metabolomics in urine samples from epidemiological studies would require large sample sizes to detect associations with moderate effect sizes.

## Introduction

A metabolome refers to the collection of low-molecular-weight compounds that form the intricate biochemical network in an organism [1]. Over the past decade, metabolomics, the profiling of a metabolome within a given a biological specimen [2], has become a growing means to identify biomarkers that can measure environmental exposures [3], assess disease risk [4], [5], and diagnose diseases at an early stage [6], [7]. While these initial studies have demonstrated the potential of metabolomics, several important issues need to be resolved before considering metabolomics as a tool for population-based research.

In a large epidemiological study, the aim is to identify metabolites that are associated with disease risk or exposure status. Researchers are primarily interested in the “usual” metabolite level, which can be loosely translated as the average level over a period of time. The presumption is that the average metabolite levels are most likely to be indicative of disease risk or exposure. However, the human metabolome is highly dynamic. Some metabolites may fluctuate with internal rhythms, such as circadian or lunar rhythms [8]–[10]. Others may vary in response to external stimuli such as foods, drugs, and other environmental exposures [11], [12], and others might change with long-term secular trends. A single measure, which may be all that is available in some epidemiological studies, may not capture the usual level and associations may be more difficult to detect [13], [14]; therefore, it is important to evaluate the contribution of within-person variability, in addition to technical variability or measurement error, to the total variation of metabolites in a study population. Such information would allow researchers to determine whether an epidemiological study will have the power to detect associations, and if so, to help optimize the study design.

The two biospecimens commonly collected for studies are blood and urine. Recently several studies have examined the reliability of metabolites in serum and plasma [14]–[16]. The studies by Floegel et al. [15], Kotsopoulos et al. [16] and by Townsend et al. [17] reported, on average, moderate stability over time in serum and plasma metabolites, as judged by an intraclass-correlation coefficient (ICC, median value 0.4∼0.5) covering several months to a year. In a third study, we used a non-targeted approach using serum samples and calculated 1-year 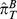, a measure that estimates the proportion of total variance attributable to between-individual variance and is similar to ICCs reported in previous studies. Our findings were largely similar with a median 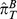 of 0.43[14]. Unfortunately, similar studies on urinary metabolites have been limited, and they have focused on a relatively limited number of compounds or classes of compounds, such as phytoestrogens [16] and phthalates [18], or on specific exposures such as estrogen usage [19], nicotine [20] and polycyclic aromatic hydrocarbons [21].

We extend our previous investigation of sources of variability in serum metabolites by studying a large set of 539 metabolites measured in urine. Our overall goal is to provide the information needed to design the metabolomic component of large-scale epidemiological studies. Our first objective is to estimate the technical, within-individual, and between-individual variability of urine metabolites. Our second objective is to translate these estimates of variability into estimates of the study power that can be expected for epidemiological studies, with a specific focus on large case-control and nested case-control studies. Although statistical considerations for case-control and nested case-control studies are identical, nested case-control studies, where biospecimen are collected prior to diagnosis, have the practical benefit of avoiding reverse causation or the detection of disease effects. We consider power as a function of the number of samples collected, sample size, and number of metabolites tested. Because metabolomics measures are relatively expensive, our power calculation also considered the alternative design of pooling multiple samples collected at different time points from an individual and running a single measurement on the pooled sample.

## Methods

### Study Population and Urinary Sample Collection

Our study of metabolite variability was performed on samples collected as part of the Navy Colon Adenoma Study, a case-control study of colorectal adenoma conducted at the National Naval Medical center; details of this study have been previously reported [22]. Cases were patients diagnosed with colorectal adenomas by sigmoidoscopy or colonoscopy between March 1993 and September 1996. Controls were selected among patients free of colorectal adenoma at sigmoidoscopy during the same time period and were frequency matched to cases on age, race and gender. The participants provided written informed consent and the study was approved by the Institutional Review Boards of both the National Cancer Institute and the National Naval Medical Center.

Cases and controls were eligible for the study if they lived within 60 miles of Washington D.C., were 18–74 years of age and had no history of Crohn’s disease, ulcerative colitis, or cancer except non-melanoma skin cancer. From the 244 cases, we selected 131 cases with no previous history of rectal bleeding or adenoma, complete questionnaire data and serum and urine samples available. An equal number of controls were selected to match the cases on age (5-year age groups), sex, and smoking status (ever or never). Participants provided information on demographics, life style, family history of cancer, occupation history and medical history.

Interviewers collected non-fasting overnight urine samples at home visits approximately 60–90 days after the screening exam. All 262 cases and controls had one urine sample collected at this first “baseline” visit, and subsequent urine samples were collected from 17 male controls. Of these 17 individuals, 12 had samples collected from a total of three home visits and 5 had samples collected from two home visits. The median time between consecutive home visits was 2 days, while the median time between the two most distant visits was 10 days. Furthermore, technical replicates were measured from one sample from each individual, with 15 samples having 2 technical replicates and 2 having 3 replicates. Urine samples were aliquoted and stored frozen.

### Metabolite Measurement

Urinary metabolites were measured by Metabolon Inc. (North Carolina, U.S.) and the platform and process have been previously described [23]. A non-targeted extraction was used, followed by protein precipitation to recover a diverse set of metabolites. Samples were then analyzed using ultra high performance liquid-phase chromatography with tandem mass spectrometry (LC-MS) and gas chromatography coupled with mass spectrometry (GC-MS). The mass spectra peaks were linked to a chemical reference library generated to identify individual metabolites and determine their relative quantities. Identified metabolites were grouped into 8 chemical classes (amino acids, carbohydrates, cofactors and vitamins, energy metabolites, lipids, nucleotide metabolites, peptides, and xenobiotics). To account for variability by run day, metabolites were individually normalized by run day and expressed in relative concentrations.

Values below the detection threshold were set to the minimum observed value of the metabolite. Metabolites that were observed in ≤90% of the samples were excluded from the analysis. We log-transformed the metabolite levels, following common practice in metabolomic studies. In sensitivity analyses, we evaluated potential confounding by sample concentration by dividing metabolite levels by sample osmolarity, a measure of solute concentration, prior to log transformation.

### Statistical Analysis

As previously described [14], we decomposed the total variance of each metabolite, 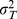, into three different components: the between subject variance, 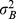, which represents the variance of the “usual” level for subjects in a population; the within subject variance, 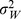, which reflects the variability over time around the “usual” level within an individual; the technical variance, 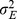, which is the variance introduced by measurement error in the laboratory procedures.

From these three variance components, we defined the following additional quantities:

1. Biological variance: the combination of between- and within-person variances, 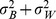.
2. Technical ICC: the proportion of the total variance that is attributed to biological variance, as opposed to random laboratory variation. High technical ICC indicates high laboratory reproducibility.

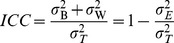

3. 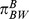, the proportion of the population’s biological variance that is attributed to between-individual variance.

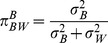

4. 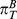, the proportion of the total variance that is attributed to between-individual variance. Higher 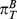 will likely indicate higher power to detect associations.

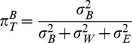
To estimate 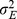, 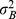, 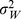 and relevant quantities, we assumed that the log-transformed metabolite level, Y_ij_, for subject i on day j could be described by the following linear mixed model [24]

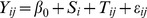
(1)where Si is a subject-specific random effect with 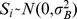, Tij is a time-specific random effect 
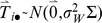
, where Σ is the correlation matrix that reflects either independence (Σij = 1 if i = j, 0 otherwise) or autocorrelation (Σ_ij_ = ρ^|i–j|^), and εij is measurement error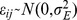. Here, “
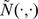
” indicates that a variable follows a normal distribution and ρ represents the correlation between levels one day apart.We further assumed that S, T, and ε are independent. Therefore, letting 1 denote a matrix with every entry equal to one and letting I denote the identity matrix, we let



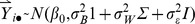



For each metabolite, we fit one model assuming the covariance matrix, Σ, has an autocorrelation structure and one model assuming that Σ has an independence structure. We compared the two models and tested for the existence of correlation over time (e.g. ρ≠0) by the Lagrange multiplier test [17] performed by the plm package in R [25]. Because of the small number of replicate samples, our primary analyses will use the estimates of 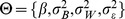
from the smaller model assuming Σ is the identity matrix.

### Evaluating Age, Gender and BMI

Age, gender and body mass index (BMI) are among the commonly adjusted characteristics in epidemiologic studies, and these factors may be related to metabolite levels [26], [27]. As previously described [14], we examined the variance of each metabolite attributable to these factors by expanding equation (1) to include fixed effects for age (quartiles), gender (male and female) and BMI (<25, 25–29.9, and ≥30 kg/m^2^).
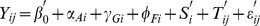
(2)Where 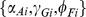 are the age, gender, and BMI effects for individual i, and 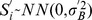, 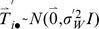, and 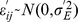.

The total variance is now defined by
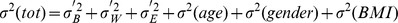
The adjusted 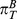, or 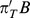, is defined as 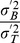.

We further denoted the proportion of the total variance attributable to age, gender and BMI as π(age), π(gender) and π(BMI).
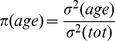


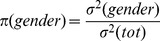


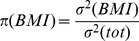



We also assessed whether the covariates were significantly associated with metabolite levels and obtained p-values by performing an analysis of variance on these mixed models.

### Power

We estimated the expected power for a case-control study focused on a single outcome using previously described methods [14]. Specifically, we assume that a study will collect *n* individuals with an 1∶1 ratio of cases:controls. We consider other ratios, 2∶1 and 3∶1, in the supporting information. We further assume that the study will use a *t* test to compare the metabolite levels between cases and controls to detect associations between metabolites and disease, using a Bonferroni-corrected significance threshold. We defined the effect size as the relative risk (RR) of disease comparing individuals in the top to the bottom quartiles of the “usual” metabolite level. At a given effect size, we calculated, across metabolites, the mean probability of detecting a statistically significant association, accounting for the 3 sources of variability. This average probability, or the average power, indicates the proportion of true metabolite-disease associations that we expect to discover in a given study. We calculated power for studies that have different number of participants and different α-levels. We also considered the scenario where multiple samples were taken from each individual at different times, but pooled together so that only a single (and potentially expensive) laboratory measurement would be needed for the mixed solution. In this scenario, the power calculation is based on 1/x of the within-individual variability (x = number of samples per person). Finally, we estimated the expected power when a more liberal cutoff, based on the False Discover Rate (FDR), replaced the Bonferroni-corrected threshold. A significance threshold based on FDR depends on the number and effect sizes of the metabolites truly associated with the outcome. For our example, we considered an outcome similar to BMI, and calculated the p-value thresholds that corresponded to FDRs of 0.05, 0.1, and 0.2 using the distribution of p-values from testing associations with BMI.

## Results

### Laboratory/Technical Variability

**Figure 1:**
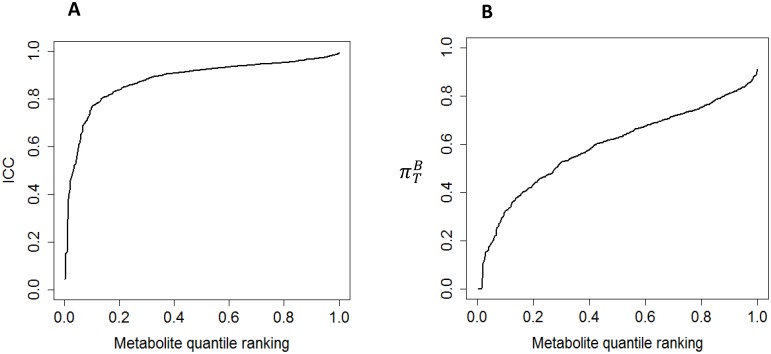
The plots illustrate the distribution of technical ICCs (A) and 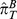 (B) of overnight urinary samples in the Navy Colon Adenoma Study. The ICC is a measure of laboratory variability. The 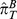 is a measure of between-individual variance. The curves illustrate the ICC and 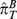 for the specified metabolite quantile ranking. Median ICC: 0.92. Median 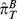: 0.62.

We detected a total of 846 metabolites in urine, with 539 being present in more than 90% of the samples. Of these 539 metabolites which were included in our analysis, 239 had a confirmed identity. Overall, laboratory reproducibility was high (**figure 1A**) with the technical ICCs for 99%, 97% and 87% of the metabolites exceeding 0.2, 0.5 and 0.8, respectively (**table 1**). The distribution of ICCs was similar across different categories of metabolites (**table S1 in File S1**). In sensitivity analyses in which we adjusted for osmolarity to control for urine sample concentration, ICCs were generally similar, with no net improvement. Our primary analyses are therefore based on models without adjustment for osmolarity.

**Table 1:** Percentage of metabolites exceeding parameter thresholds^a^ in the Navy Colon Adenoma Study.

	Parameter threshold		
	0.2	0.5	0.8
ICC^b^	99%	97%	87%
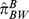 c	97%	81%	31%
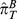 d	95%	72%	12%

a Each row list the percentage of metabolites with an estimated parameter (ICC, 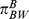 and 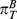) exceeding the threshold of 0.2, 0.5 and 0.8.

b ICC represents the proportion of total variation attributable to biological variance.

c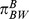 represents the proportion of biological variability attributable to between-individual variance.

d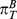 represents the proportion of total variation attributable to between-individual variance.

### Within and between-individual Variability

Two sources of variability–within and between-individual variance–constitute the total biological variability. For most of the metabolites, between-individual variability explains the majority of the total variance: 72% of the metabolites had a 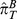 exceeding 0.5 (**table 1**). However, only 12% of the metabolites had a 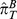 higher than 0.8. A list of identified metabolites with the highest 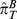 is presented in **table 2**. Among different metabolite categories, amino acid, lipid and xenobiotics had the highest proportion of metabolites with 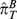 exceeding 0.8 (20%, 18% and 13%, respectively, **table S2 in File S1**). The metabolites with the lowest 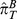 formed a heterogeneous group and were involved in various biochemical pathways (**table S3 in File S1**). A full list of 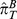 as well as ICCs of all identified metabolites is presented in **table S4 in File S1**.

**Table 2:** A list of identified metabolites with the highest values of between subject variability, 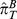 (e.g., the lowest within-subject variability), among all metabolites in the Navy Colon Adenoma Study.

Metabolite	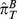	Category	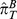 , adjusted^a^	*P* value gender	*P* value age	*P* value BMI
androsterone sulfate	0.90993	Lipid	0.89726	0.00060	<.0001	0.3118
pregnen-diol disulfate	0.89928	Lipid	0.87069	<.0001	<.0001	0.1976
3-aminoisobutyrate	0.88697	Nucleotide	0.88400	0.56600	0.00840	0.5058
1,7-dimethylurate	0.88549	Xenobiotics	0.88348	0.02520	0.73660	0.1966
tryptophan betaine	0.87904	Amino acid	0.87510	0.00270	0.47490	0.4873
N-acetyl-beta-alanine	0.87158	Amino acid	0.86605	0.89760	0.26440	0.842
N-acetylasparagine	0.86819	Amino acid	0.86979	0.25490	0.73320	0.5917
pantothenate	0.86148	Cofactors and vitamins	0.85960	0.00840	0.15100	0.4977
glucose	0.85992	Carbohydrate	0.85168	0.18910	0.17480	0.7651
fucose	0.85541	Carbohydrate	0.85024	0.12220	0.00460	0.001
paraxanthine	0.85534	Xenobiotics	0.85197	0.05710	0.17840	0.1712
glutaroyl carnitine	0.85349	Amino acid	0.83457	<.0001	<.0001	0.0863
4-androsten-3beta,17beta-diol disulfate 2	0.85143	Lipid	0.79376	0.00030	<.0001	0.0422
andro steroid monosulfate 1	0.85110	Lipid	0.80648	<.0001	<.0001	0.1097
5-acetylamino-6-amino-3-methyluracil	0.84867	Xenobiotics	0.84641	0.02400	0.39010	0.8002
N-acetyltyrosine	0.84795	Amino acid	0.84868	0.88960	0.02810	0.0108
glycylproline	0.84599	Peptide	0.83253	0.03260	<.0001	0.3464
phenylalanine	0.84218	Amino acid	0.82608	0.00890	0.02410	0.0004
stachydrine	0.83748	Xenobiotics	0.84008	0.69140	0.86810	0.7278
citrate	0.83653	Energy	0.83598	0.28410	0.16060	0.0898
3-indoxyl sulfate	0.83496	Amino acid	0.83718	0.31590	0.62640	0.0338
serine	0.83040	Amino acid	0.81117	0.00030	0.00040	0.1435
creatinine	0.82840	Amino acid	0.79386	<.0001	0.00010	0.1667
methylglutaroylcarnitine	0.82539	Amino acid	0.82424	0.06120	0.86790	0.0883
tyrosine	0.81789	Amino acid	0.79326	0.00100	0.43500	<.0001
tryptophan	0.81691	Amino acid	0.79820	0.00340	0.02510	<.0001
N-acetylglutamine	0.81477	Amino acid	0.81638	0.93390	0.36430	0.3209
indolelactate	0.81370	Amino acid	0.81769	0.78720	0.80910	0.6973
glycocholenate sulfate	0.81102	Lipid	0.80034	0.01020	0.14040	0.0009
lysine	0.80945	Amino acid	0.79957	0.46750	0.14440	0.0003
N4-acetylcytidine	0.80942	Nucleotide	0.79722	0.00110	0.10460	0.0156
3,4-dihydroxyphenylacetate	0.80810	Amino acid	0.80567	0.50410	0.00950	0.77
kynurenine	0.80704	Amino acid	0.79518	0.39490	0.01820	<.0001
glutamine	0.80483	Amino acid	0.78034	0.02310	0.00020	0.6464
dimethylglycine	0.80274	Amino acid	0.78929	0.00910	0.36200	0.0001

a adjusted for age (quartiles), gender (male, female) and BMI (<25, 25–<30 and 30+kg/m^2^).

We also evaluated 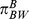, the proportion of biological variability attributed to between-individual variance. The majority of metabolites had a relatively high 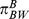, with 97%, 81% and 31% having a 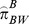 exceeding 0.2, 0.5 and 0.8, respectively (**table 1**).

**Figure 2:**
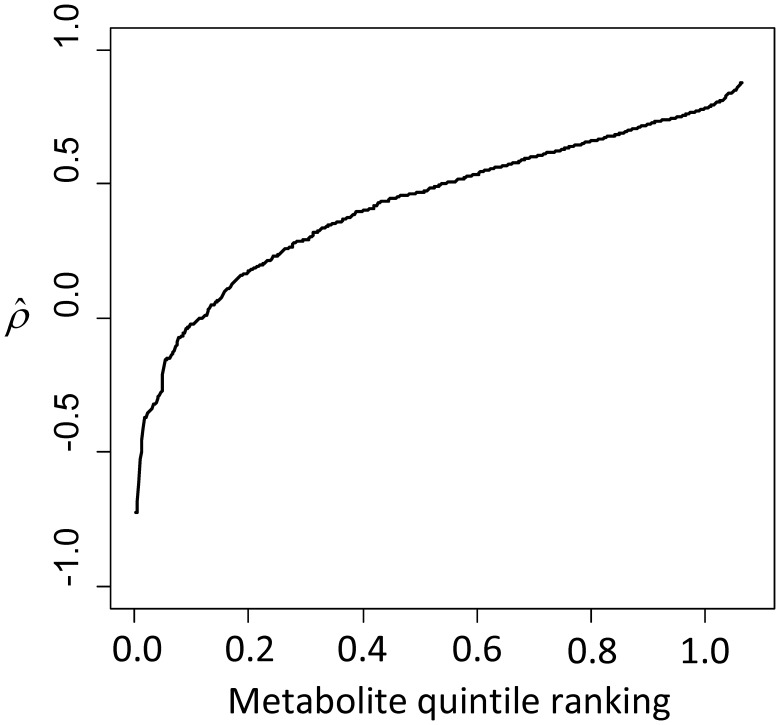
The plot illustrates the distribution of 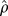, an estimate of a measure of autocorrelation over time, for all metabolites. The curve illustrates that the majority of ρ are likely above 0 and that measurements collected on consecutive days are likely more similar than those collected one week apart. The x-axis indicates the quantile ranking and y-axis indicates 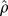 for a metabolite at that ranking. For example, the median level, that of the metabolite ranked 284, is 0.49.

Of the 41 pairs of samples taken at different time points from the same individual, we had 3, 14, 8, 5, 5, 2, 1, 1, and 2 pairs separated by 1, 2, 3, 4, 5, 6, 7, 8 and 10 day intervals, respectively. To evaluate whether pairs separated by shorter intervals were more highly correlated, we estimated ρ from the linear mixed models described by equation (1) with ∑ assumed to have an autocorrelation structure, and obtained a p-value evaluating whether the autocorrelation structure was a significant improvement over independence. The distribution of ρ (**figure 2**) and p-values (**figure S1 in File S1**) suggested that the correlation between measurements within an individual decreased slightly with the time even over this 1 week. However, our study did not have sufficient power to precisely estimate this correlation, and using a Bonferroni corrected threshold of 0.05/539, could only find 7 metabolites (orotidine, andro steroid monosulfate and five unidentified metabolites) with a ρ statistically significantly larger than 0.

### Age, Gender and BMI

**Figure 3:**
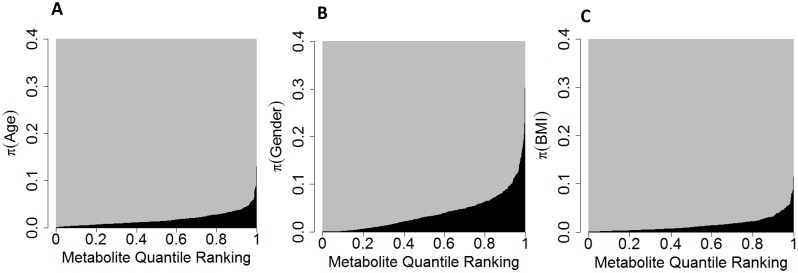
The distribution of 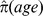
(A), 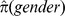
(B), and 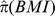
(C). The x-axis represents the metabolite quantile ranking, and the y-axis represents 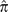. The black areas under the curve illustrate the for the specified metabolite quantile ranking, which shows the variance explained by these three covariates.

Age, gender and BMI explained only a small proportion of the total variation (**figure 3**). Moreover, we found that these covariates were only significantly associated with small percentages of metabolites. Using the Bonferroni-adjusted α-level of 0.05/539, we found 1.3%, 12.4%, and 0.7% of the metabolites were significantly associated with age, gender and BMI, respectively.

Epidemiologic studies often adjust for potential confounders. In this case, the study power is reflected in 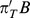, or the adjusted 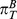, which represents the proportion of total variability attributed to between-individual variance after adjusting for age, gender and BMI. Overall we found only small differences between 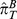 and 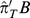. The percentages of metabolites with 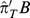 exceeding 0.2, 0.5, and 0.8 were 94%, 70% and 9.7%, respectively. Among metabolites with the highest ratio of between-individual to total variance (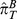), including age, gender and BMI in the regression model had little impact on the estimation of 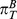 (**table 2**).

### Power

**Figure 4:**
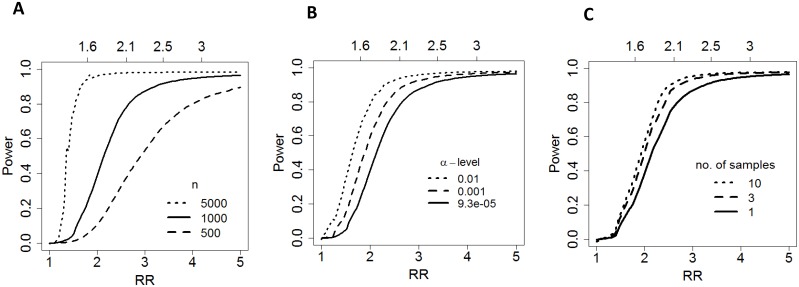
The curves show the proportion of metabolites expected to be detected in a case control study as a function of effect size. Effect size is defined by the relative risk (RR, on the x-axis) of disease comparing individuals in the top and bottom quartiles of the “usual” metabolite level. The top axis shows the naïve relative risk that would be observed in the study without adjusting for measurement error. Each figure varies one parameter: sample size, α-level, or the number of samples/individual. (A) presents power curves according to different sample size (n of 500, 1,000 and 5,000) under a Bonferroni-adjusted α-levels (0.05/539); (B) presents power curves with different α-levels in a case-control study of 1,000 individuals; (C) presents power curves in a case-control study of 1,000 individuals, with different number of distinct urinary samples (1, 3, and 10, α-level = 0.05/539).

With the observed technical, within-individual and between-individual variances, we estimated study power to detect an RR of 1.5, 3.0 and 5.0 in a case-control design using a Bonferonni adjusted α-level of 0.05/539. We found that a study with 500 individuals is expected to detect 1%, 54% and 89% of the metabolites with a true RR of 1.5, 3.0 and 5.0, respectively. In a study of 1,000 individuals, we expect the proportions of metabolites detected at these RR increase to 4.5%, 88% and 96%. With a sample size of 5,000, we expect to detect 75% of the metabolites even at a RR of 1.5 (**table 3** **,** **figure 4A**) and approximately 100% of metabolites with a RR of 3.0 and 5.0. The impact of 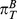 on study power is highlighted in **figure S2 in File S1**, where we show, for example, that the power to detect a metabolite with a RR of 2.0 would decrease from 0.8 to 0.3 as 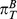 decreases from 1 to 0.5. Without correcting for measurement error, the observed RR reported in an epidemiologic study would be attenuated. Therefore we defined the naïve RRs as the RR that would be estimated based on the observed metabolite measurements. We have estimated that, given the within-individual variance observed in our study, when true RRs are 1.5, 3.0 and 5.0, the uncorrected, naïve RRs are expected to be 1.4, 2.3 and 3.4, respectively.

**Table 3:** Average study power to detect associations between metabolites and disease in a case-control study according to different relative risks and sample sizes.

	True relative risk^a^		
Sample size (1∶1 case:control ratio)	1.5	3.0	5.0
500	1.0%	54%	89%
1,000	4.5%	88%	96%
5,000	75%	98%	98%

a The naïve estimates of true relative risks of 1.5, 3.0 and 5.0 would be 1.4, 2.3 and 3.4, respectively.

In metabolomics studies with a targeted approach, researchers can restrict the analysis to a predetermined list of metabolites based on *a priori* knowledge. In this scenario, the number of metabolites being evaluated may be substantially smaller, resulting in a higher α-level, due to a smaller penalty from the Bonferroni correction. We examined power curves with different α-levels (**figure 4B**). When we raise the α-level from 0.05/539 to 0.001, a case-control study with 1,000 individuals is expected to detect of 13.5%, 93% and 98% of the metabolites with a true RR of 1.5, 3.0 and 5.0, respectively. We considered more liberal p-value thresholds of significance, 0.0096, 0.0226 and 0.0660, corresponding to FDRs of 0.05, 0.1, and 0.2 (**figure S3A in File S1**). In the most liberal scenario, with an FDR = 0.2, a case-control study with 1,000 individuals is expected to detect of 59%, 97%, and 98% of the metabolites with a true RR of 1.5, 3.0 and 5.0.

We evaluated how collecting multiple samples would help to improve study power. We assume that a study will collect up to 10 urinary samples from each individual and mix these samples to perform one measurement of metabolite levels on the pooled sample. This approach will reduce within-individual variance and better capture an individual’s usual metabolite level. As shown in **figure 4C**, collecting multiple samples would only slightly improve study power. By collecting 3 samples per person on a case-control study of 1,000 individuals, we expect to increase the study power by 1.44x, 1.11x and 1.02x with a true RR of 1.5, 3.0 and 5.0, respectively, which would mean an average probability of detecting 6.7%, 93% and 98% of the metabolites. We also estimated that even when each one of the multiple samples is individually measured, the improvement in statistical power would be limited with increasing numbers of samples (data not shown), which is what would be expected when technical error is low. Finally, we examined the influence of case:control ratio, at a given study sample size, on study power. We found that comparing to a 1∶1 ratio, increasing the control-to-case ratio to 3∶1 would only improve study power slightly (**figure S3B in File S1**).

## Discussion

We investigated the source of variability of 539 metabolites measured by LC-MS and GS-MS in urine samples from the Navy Colon Adenoma Study. We found that overall technical reliability was high, and for most of the metabolites, the majority of total variance can be attributed to between-individual variability. However, despite relatively high 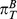, we estimated that large sample sizes (hundreds to thousands of participants) would be needed for case-control and nested case-control studies to detect metabolite associations with moderate effect sizes (RR of 1.5–3.0).

In an epidemiological study, the degree to which one or a few measures can accurately assess an individual’s “usual” level is a key determinant of statistical power. With the same effect size for usual level, it is easier to detect associations for metabolites that have low variability over time and minimal measurement error. Consistent with our findings, a previous study that examined the metabolic profile of over 700 unique metabolite peaks from serum and urine samples using GC-MS reported much smaller variance among technical replicates, compared with variance among different individuals [28]. Similarly high reproducibility has also been observed in studies examining LC-MS methods for global metabolic profiling in urine [29]. Although technical improvements will lead to more accurate measures of metabolites, the high reproducibility of current laboratory methods suggests that in order to enhance the power to detect metabolite associations, other approaches, such as increasing sample size or making multiple measurements, must also be considered when metabolomic studies are planned.

The levels and patterns of temporal variability differ by metabolites. Some metabolites, such as cortisol and melatonin, fluctuate by the hour in a given day [9], [28]; while other metabolites, such as female hormones and vitamin D, may fluctuate by month or season. To examine associations with these metabolites, studies should either try to collect specimens at similar times for all subjects or record the time so that downstream analyses can perform appropriate adjustments. Another source of variability is time since exposure. For example, markers of diet, smoking, alcohol consumption and use of medication may be heavily influenced by the most recent exposure. In this case, researchers may consider making repeated measures, as this sampling practice can enhance the power of detection and benefit epidemiological studies that involve exposure assessment using biosamples [11]. When resources are limited and do not permit making repeated measures on the entire study population, it may still be beneficial to obtain multiple measurements on a subsample to allow estimation of within-individual variation. On the other hand, as suggested by our power calculation, when within-individual variance is relatively low, the most effective way to increase detection power may be expanding the study population.

In previous studies of serum metabolites, blood samples were collected over a few months to several years [14]–[16]. In contrast, in this study, the time intervals between sample collections were relatively short, spanning from 1 to 10 days. This distinction may partially explain why we observed a higher 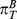
of urinary metabolites when compared with the serum findings in our previous study [14](mean: 0.60 for urine vs. 0.43 for serum). For many metabolites, the correlation between samples would be expected to be higher when time intervals between sample collections are short. As a result, measures over a few days may not capture the true temporal variability around the “usual” level of these metabolites.

Of the total variance in urinary metabolite levels, only a small proportion overall was explained by age, BMI, and gender. For gender, there were a substantial number of associations–over 10% of metabolites were associated with gender; however, many of them were metabolites of sex hormones that are usually examined separately for males and females in epidemiological studies regardless. Factors besides age, BMI, and gender likely explain the remaining variance, and thus there is high potential for discovery of new exposures of interest for disease in epidemiologic studies.

There are several limitations of our study. First, we calculated technical and within-individual variance based on only 37 and 46 samples, respectively. This may lead to imprecise estimation of 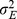 and 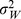, and other related quantities for specific metabolites. However, we focus on the distributions of these quantities across all metabolites, and these distributions should be relatively accurate. Additionally, due to the small sample size of non-technical replicates, our study was underpowered to detect temporal autocorrelation among metabolites. Another limitation is that the relatively short time interval separating sample collections may lead to underestimation of within-individual variability over the long term. Finally, our power calculation considered only the detection of individual metabolite and disease associations, but did not assess the power for identifying metabolomic profiles associated with disease.

In summary, we evaluated the variability of urinary metabolites from three sources and estimated the power of case-control studies based on our findings. We showed that our previously developed framework is useful in determining sample sizes when metabolomic studies are planned.

## Supporting Information

File S1 **Supporting figures and tables.**
(DOCX)
