# Supplementary material for: Sources of Variability in Metabolite Measurements from Urinary Samples
Source: PLoS One. 2014 May 1;9(5):e95749. doi: 10.1371/journal.pone.0095749 (PMC4006796; doi:10.1371/journal.pone.0095749)
Supplement: File S1 — Supporting figures and tables. (DOCX) [file pone.0095749.s001.docx]

Frequency

**Figure S1.** A histogram of the p-values from the Lagrange multiplier test of the null hypothesis that ρ = 0.


$$\pi_{T}^{B}$$

Power

**Figure S2.** The curves show the proportion of metabolites expected to be detected in a case control study (n=1,000) as a function of $\pi_{T}^{B}$, according to different relative risks.

**B**

**A**

**Figure S3.** The curves show the proportion of metabolites expected to be detected in a case control study as a function of effect size. Each power curve in **A)** represents a different false discovery rate (0.05, 0.1, 0.2, corresponding to α-levels of 0.0096, 0.0226, and 0.0660, respectively, based on our results on BMI association with metabolites); and in **B)** represents a different case-to-control ratio (1:1, 1:2, and 1:3), with α-level=0.05/539. Total sample size: 1,000.

**Table S1** Percentage of metabolites exceeding ICC thresholds in the Navy Colon Adenoma Study, by metabolite categories

|  |  | ICC threshold | | |
| --- | --- | --- | --- | --- |
| Category | N | 0.2 | 0.5 | 0.8 |
| Amino acid | 95 | 100% | 97% | 85% |
| Carbohydrate | 28 | 100% | 100% | 89% |
| Cofactors and vitamins | 13 | 100% | 92% | 92% |
| Energy | 13 | 92% | 92% | 62% |
| Lipid | 28 | 100% | 93% | 89% |
| Nucleotide | 21 | 100% | 100% | 86% |
| Peptide | 10 | 100% | 90% | 80% |
| Xenobiotics | 30 | 100% | 100% | 93% |
|  |  |  |  |  |

**Table S2** Percentage of metabolites exceeding $\pi_{T}^{B}$ thresholds in the Navy Colon Adenoma Study, by metabolite categories

|  |  | $\pi_{T}^{B}$ threshold | | |
| --- | --- | --- | --- | --- |
| Category | N | 0.2 | 0.5 | 0.8 |
| Amino acid | 95 | 95% | 81% | 20% |
| Carbohydrate | 28 | 93% | 64% | 7% |
| Cofactors and vitamins | 13 | 100% | 62% | 8% |
| Energy | 14 | 85% | 62% | 8% |
| Lipid | 28 | 100% | 82% | 18% |
| Nucleotide | 21 | 95% | 86% | 10% |
| Peptide | 10 | 80% | 60% | 10% |
| Xenobiotics | 30 | 93% | 73% | 13% |
|  |  |  |  |  |

**Table S3** A list of identified metabolites with the lowest $\hat{\pi}_{T}^{B}$ among all metabolites in the Navy Colon Adenoma Study

| **Metabolite** | ${\hat{\boldsymbol{\pi}}}_{\boldsymbol{T}}^{\boldsymbol{B}}$ | **Category** | **Pathway** | ${\hat{\boldsymbol{\pi}}}_{\boldsymbol{T}}^{\boldsymbol{B}}$**, adjusted ^a^** | ***P* value gender** | ***P* value age** |
| --- | --- | --- | --- | --- | --- | --- |
| 1,6-anhydroglucose | 0.19190 | Carbohydrate | Glycolysis, gluconeogenesis, pyruvate metabolism | 0.00000 | 0.23460 | 0.38690 |
| succinate | 0.18502 | Energy | Krebs cycle | 0.24331 | 0.28420 | 0.21020 |
| tartarate | 0.17838 | Xenobiotics | Food component/Plant | 0.13762 | 0.12930 | 0.34840 |
| allantoin | 0.17496 | Nucleotide | Purine metabolism, urate metabolism | 0.14849 | 0.02310 | 0.00520 |
| itaconate (methylenesuccinate) | 0.15490 | Energy | Krebs cycle | 0.12891 | 0.06350 | 0.81130 |
| pyroglutamylvaline | 0.15224 | Peptide | Dipeptide | 0.11205 | 0.00590 | 0.82460 |
| 2-oxindole-3-acetate | 0.00000 | Xenobiotics | Food component/Plant | 0.00000 | 0.82870 | 0.10880 |
| xylose | 0.00000 | Carbohydrate | Nucleotide sugars, pentose metabolism | 0.00000 | 0.81940 | 0.83920 |
| phenol sulfate | 0.00000 | Amino acid | Phenylalanine & tyrosine metabolism | 0.00000 | 0.87320 | 0.30920 |
| glycylglycine | 0.00000 | Peptide | Dipeptide | 0.00000 | 0.00940 | 0.75830 |

^a^ adjusted for age (quartiles), gender (male, female) and BMI (<25, 25-<30 and 30+ kg/m^2^)

**Table S4** $\hat{\pi}_{T}^{B}$ and ICCs of identified metabolites in the Navy Colon Adenoma Study

| **metabolite** | ${\hat{\boldsymbol{\pi}}}_{\boldsymbol{T}}^{\boldsymbol{B}}$ | **ICC** | **Category** |
| --- | --- | --- | --- |
| androsterone sulfate | 0.91 | 0.95 | Lipid |
| pregnen-diol disulfate* | 0.90 | 0.90 | Lipid |
| 3-aminoisobutyrate | 0.89 | 0.95 | Nucleotide |
| 1,7-dimethylurate | 0.89 | 0.95 | Xenobiotics |
| tryptophan betaine | 0.88 | 0.96 | Amino acid |
| N-acetyl-beta-alanine | 0.87 | 0.93 | Amino acid |
| N-acetylasparagine | 0.87 | 0.88 | Amino acid |
| pantothenate | 0.86 | 0.95 | Cofactors and vitamins |
| glucose | 0.86 | 0.95 | Carbohydrate |
| fucose | 0.86 | 0.97 | Carbohydrate |
| paraxanthine | 0.86 | 0.95 | Xenobiotics |
| glutaroyl carnitine | 0.85 | 0.94 | Amino acid |
| 4-androsten-3beta,17beta-diol disulfate 2* | 0.85 | 0.91 | Lipid |
| andro steroid monosulfate 1* | 0.85 | 0.86 | Lipid |
| 5-acetylamino-6-amino-3-methyluracil | 0.85 | 0.90 | Xenobiotics |
| N-acetyltyrosine | 0.85 | 0.95 | Amino acid |
| glycylproline | 0.85 | 0.92 | Peptide |
| phenylalanine | 0.84 | 0.91 | Amino acid |
| stachydrine | 0.84 | 0.95 | Xenobiotics |
| citrate | 0.84 | 0.95 | Energy |
| 3-indoxyl sulfate | 0.83 | 0.97 | Amino acid |
| serine | 0.83 | 0.91 | Amino acid |
| creatinine | 0.83 | 0.95 | Amino acid |
| methylglutaroylcarnitine | 0.83 | 0.92 | Amino acid |
| tyrosine | 0.82 | 0.86 | Amino acid |
| tryptophan | 0.82 | 0.87 | Amino acid |
| N-acetylglutamine | 0.81 | 0.90 | Amino acid |
| indolelactate | 0.81 | 0.93 | Amino acid |
| glycocholenate sulfate* | 0.81 | 0.91 | Lipid |
| lysine | 0.81 | 0.94 | Amino acid |
| N4-acetylcytidine | 0.81 | 0.89 | Nucleotide |
| 3,4-dihydroxyphenylacetate | 0.81 | 0.88 | Amino acid |
| kynurenine | 0.81 | 0.95 | Amino acid |
| glutamine | 0.80 | 0.91 | Amino acid |
| dimethylglycine | 0.80 | 0.94 | Amino acid |
| 21-hydroxypregnenolone disulfate | 0.80 | 0.95 | Lipid |
| trans-urocanate | 0.80 | 0.87 | Amino acid |
| creatine | 0.79 | 0.82 | Amino acid |
| scyllo-inositol | 0.79 | 0.90 | Lipid |
| ethanolamine | 0.79 | 0.79 | Lipid |
| gluconate | 0.79 | 0.95 | Carbohydrate |
| adenosine 3',5'-cyclic monophosphate (cAMP) | 0.79 | 0.95 | Nucleotide |
| alpha-CEHC glucuronide* | 0.79 | 0.97 | Cofactors and vitamins |
| N6-acetyllysine | 0.78 | 0.88 | Amino acid |
| leucine | 0.78 | 0.94 | Amino acid |
| methionine | 0.78 | 0.91 | Amino acid |
| N2,N2-dimethylguanosine | 0.78 | 0.92 | Nucleotide |
| quinate | 0.77 | 0.94 | Xenobiotics |
| N-acetylhistidine | 0.77 | 0.91 | Amino acid |
| sarcosine (N-Methylglycine) | 0.77 | 0.93 | Amino acid |
| succinylcarnitine | 0.76 | 0.97 | Energy |
| homovanillate (HVA) | 0.76 | 0.91 | Amino acid |
| pseudouridine | 0.76 | 0.97 | Nucleotide |
| ribitol | 0.76 | 0.91 | Carbohydrate |
| gamma-glutamylleucine | 0.76 | 0.92 | Peptide |
| 1-methylxanthine | 0.76 | 0.89 | Xenobiotics |
| 3-methylglutaconate | 0.75 | 0.92 | Amino acid |
| erythronate* | 0.75 | 0.93 | Carbohydrate |
| N-acetylputrescine | 0.74 | 0.90 | Amino acid |
| threonine | 0.74 | 0.91 | Amino acid |
| 4-imidazoleacetate | 0.74 | 0.92 | Amino acid |
| N-acetylarginine | 0.74 | 0.85 | Amino acid |
| neopterin | 0.74 | 0.89 | Nucleotide |
| valine | 0.74 | 0.89 | Amino acid |
| beta-hydroxyisovalerate | 0.74 | 0.93 | Amino acid |
| 2-aminobutyrate | 0.73 | 0.90 | Amino acid |
| hydroxyisovaleroyl carnitine | 0.73 | 0.95 | Amino acid |
| N6-carbamoylthreonyladenosine | 0.73 | 0.95 | Nucleotide |
| N-acetylisoleucine | 0.73 | 0.92 | Amino acid |
| p-cresol sulfate | 0.73 | 0.99 | Amino acid |
| sorbitol | 0.73 | 0.97 | Carbohydrate |
| alanine | 0.73 | 0.94 | Amino acid |
| myo-inositol | 0.73 | 0.90 | Lipid |
| dimethylarginine (SDMA + ADMA) | 0.73 | 0.91 | Amino acid |
| ornithine | 0.73 | 0.73 | Amino acid |
| glycolithocholate sulfate* | 0.73 | 0.93 | Lipid |
| 3-hydroxy-2-ethylpropionate | 0.73 | 0.94 | Amino acid |
| 2-hydroxyglutarate | 0.72 | 0.90 | Lipid |
| phenylacetylglutamine | 0.72 | 0.97 | Amino acid |
| hippurate | 0.72 | 0.96 | Xenobiotics |
| nicotinate | 0.72 | 0.89 | Cofactors and vitamins |
| vanillylmandelate (VMA) | 0.72 | 0.93 | Amino acid |
| 5-oxoproline | 0.72 | 0.96 | Amino acid |
| 3-sialyllactose | 0.72 | 0.84 | Carbohydrate |
| N2-acetyllysine | 0.72 | 0.90 | Amino acid |
| N1-methyladenosine | 0.72 | 0.94 | Nucleotide |
| 4-ureidobutyrate | 0.72 | 0.89 | Nucleotide |
| 2-methylcitrate | 0.72 | 0.89 | Energy |
| proline | 0.72 | 0.90 | Amino acid |
| isoleucine | 0.71 | 0.95 | Amino acid |
| cinnamoylglycine | 0.71 | 0.91 | Xenobiotics |
| N-(2-furoyl)glycine | 0.71 | 0.97 | Xenobiotics |
| pyridoxate | 0.70 | 0.89 | Cofactors and vitamins |
| 1,3,7-trimethylurate | 0.70 | 0.77 | Xenobiotics |
| cis-aconitate | 0.70 | 0.89 | Energy |
| glycerol 3-phosphate (G3P) | 0.70 | 0.92 | Lipid |
| 3-(4-hydroxyphenyl)lactate | 0.70 | 0.94 | Amino acid |
| isobutyrylglycine | 0.70 | 0.92 | Amino acid |
| orotidine | 0.70 | 0.70 | Nucleotide |
| 1-methylurate | 0.69 | 0.97 | Xenobiotics |
| 5-methylthioadenosine (MTA) | 0.69 | 0.90 | Amino acid |
| xylitol | 0.69 | 0.91 | Carbohydrate |
| glycolate (hydroxyacetate) | 0.69 | 0.93 | Xenobiotics |
| orotate | 0.69 | 0.81 | Nucleotide |
| N-acetylvaline | 0.69 | 0.85 | Amino acid |
| glucuronate | 0.69 | 0.92 | Carbohydrate |
| glycerol | 0.68 | 0.85 | Lipid |
| 7-methylguanine | 0.68 | 0.87 | Nucleotide |
| homovanillate sulfate | 0.68 | 0.77 | Amino acid |
| tiglyl carnitine | 0.68 | 0.93 | Amino acid |
| 4-acetamidobutanoate | 0.68 | 0.94 | Amino acid |
| thymine | 0.67 | 0.84 | Nucleotide |
| 2-hydroxyisobutyrate | 0.67 | 0.91 | Amino acid |
| taurine | 0.67 | 0.93 | Amino acid |
| 3-ureidopropionate | 0.67 | 0.82 | Amino acid |
| 3-hydroxysebacate | 0.66 | 0.86 | Lipid |
| ribulose | 0.66 | 0.78 | Carbohydrate |
| 3-hydroxyphenylacetate | 0.66 | 0.84 | Amino acid |
| 3-hydroxyisobutyrate | 0.66 | 0.92 | Amino acid |
| gulono-1,4-lactone | 0.65 | 0.86 | Cofactors and vitamins |
| methyl-alpha-glucopyranoside | 0.65 | 0.81 | Xenobiotics |
| fructose | 0.65 | 0.95 | Carbohydrate |
| gamma-glutamylvaline | 0.65 | 0.92 | Peptide |
| N2-methylguanosine | 0.64 | 0.96 | Nucleotide |
| trigonelline (N'-methylnicotinate) | 0.64 | 0.97 | Cofactors and vitamins |
| 3-methylxanthine | 0.64 | 0.97 | Xenobiotics |
| lactose | 0.63 | 0.93 | Carbohydrate |
| malate | 0.63 | 0.94 | Energy |
| glucosamine | 0.63 | 0.86 | Carbohydrate |
| tigloylglycine | 0.63 | 0.88 | Amino acid |
| isovalerylglycine | 0.63 | 0.93 | Amino acid |
| cis-4-decenoyl carnitine | 0.63 | 0.95 | Lipid |
| 1,2-propanediol | 0.62 | 0.94 | Lipid |
| 3-dehydrocarnitine* | 0.62 | 0.91 | Lipid |
| C-glycosyltryptophan* | 0.62 | 0.97 | Amino acid |
| 5-hydroxyhexanoate | 0.62 | 0.90 | Lipid |
| 3-methoxy-4-hydroxyphenylglycol | 0.62 | 0.83 | Amino acid |
| carnitine | 0.62 | 0.94 | Lipid |
| 1-methylnicotinamide | 0.62 | 0.92 | Cofactors and vitamins |
| gamma-glutamylisoleucine* | 0.62 | 0.94 | Peptide |
| 4-hydroxymandelate | 0.61 | 0.94 | Xenobiotics |
| hexanoylglycine | 0.61 | 0.81 | Lipid |
| lactate | 0.61 | 0.82 | Carbohydrate |
| acetylphosphate | 0.61 | 0.61 | Energy |
| 7-methylxanthine | 0.61 | 0.94 | Xenobiotics |
| N-acetylneuraminate | 0.61 | 0.88 | Carbohydrate |
| 2-aminoadipate | 0.61 | 0.78 | Amino acid |
| inosine | 0.61 | 0.83 | Nucleotide |
| catechol sulfate | 0.61 | 0.95 | Xenobiotics |
| N-acetylthreonine | 0.61 | 0.61 | Amino acid |
| adipate | 0.60 | 0.95 | Lipid |
| riboflavin (Vitamin B2) | 0.60 | 0.84 | Cofactors and vitamins |
| isobutyrylcarnitine | 0.60 | 0.85 | Amino acid |
| adenine | 0.60 | 0.86 | Nucleotide |
| 2-methylbutyrylglycine | 0.59 | 0.81 | Amino acid |
| gamma-glutamylthreonine* | 0.59 | 0.83 | Peptide |
| 3-hydroxybutyrate (BHBA) | 0.58 | 0.97 | Lipid |
| 3-methylglutarate | 0.58 | 0.85 | Amino acid |
| isovalerylcarnitine | 0.57 | 0.78 | Amino acid |
| pro-hydroxy-pro | 0.57 | 0.95 | Peptide |
| mesaconate (methylfumarate) | 0.57 | 0.86 | Energy |
| mannose | 0.57 | 0.89 | Carbohydrate |
| arabitol | 0.56 | 0.92 | Carbohydrate |
| uracil | 0.56 | 0.86 | Nucleotide |
| xanthosine | 0.56 | 0.93 | Nucleotide |
| tyramine | 0.56 | 0.91 | Amino acid |
| N-acetylleucine | 0.55 | 0.85 | Amino acid |
| erythritol | 0.55 | 0.92 | Xenobiotics |
| homostachydrine* | 0.55 | 0.88 | Xenobiotics |
| 5-hydroxyindoleacetate | 0.55 | 0.94 | Amino acid |
| homocitrate | 0.55 | 0.55 | Energy |
| glutamate | 0.54 | 0.77 | Amino acid |
| 3-hydroxyhippurate | 0.53 | 0.95 | Xenobiotics |
| xanthurenate | 0.53 | 0.89 | Amino acid |
| xylulose | 0.53 | 0.82 | Carbohydrate |
| 4-hydroxyhippurate | 0.53 | 0.99 | Xenobiotics |
| pyroglutamine* | 0.52 | 0.92 | Amino acid |
| 3-hydroxy-3-methylglutarate | 0.52 | 0.86 | Lipid |
| theobromine | 0.51 | 0.95 | Xenobiotics |
| glycine | 0.50 | 0.50 | Amino acid |
| acetylcarnitine | 0.48 | 0.94 | Lipid |
| arabonate | 0.48 | 0.93 | Cofactors and vitamins |
| 4-hydroxyphenylacetate | 0.48 | 0.94 | Amino acid |
| N-acetylphenylalanine | 0.48 | 0.88 | Amino acid |
| xylonate | 0.48 | 0.91 | Carbohydrate |
| malonylcarnitine | 0.48 | 0.48 | Lipid |
| cyclo(gly-pro) | 0.47 | 0.66 | Peptide |
| threitol | 0.47 | 0.90 | Carbohydrate |
| 2-oxo-1-pyrrolidinepropionate | 0.46 | 0.86 | Xenobiotics |
| 4-hydroxybenzoate | 0.46 | 0.85 | Xenobiotics |
| 4-guanidinobutanoate | 0.46 | 0.97 | Amino acid |
| imidazole lactate | 0.46 | 0.46 | Amino acid |
| 4-acetylphenol sulfate | 0.44 | 0.71 | Xenobiotics |
| N-carbamoylsarcosine | 0.44 | 0.55 | Amino acid |
| 2-methylbutyroylcarnitine | 0.44 | 0.77 | Amino acid |
| suberate (octanedioate) | 0.43 | 0.82 | Lipid |
| cysteine | 0.42 | 0.77 | Amino acid |
| threonate | 0.42 | 0.93 | Cofactors and vitamins |
| fumarate | 0.42 | 0.64 | Energy |
| 3-methylhistidine | 0.42 | 0.87 | Amino acid |
| arabinose | 0.41 | 0.92 | Carbohydrate |
| anserine | 0.40 | 0.85 | Peptide |
| citramalate | 0.40 | 0.92 | Amino acid |
| azelate (nonanedioate) | 0.40 | 0.94 | Lipid |
| isocitrate | 0.40 | 0.52 | Energy |
| urate | 0.40 | 0.74 | Nucleotide |
| indoleacetate | 0.38 | 0.86 | Amino acid |
| pipecolate | 0.38 | 0.87 | Amino acid |
| kynurenate | 0.38 | 0.72 | Amino acid |
| sucrose | 0.36 | 0.94 | Carbohydrate |
| allo-threonine | 0.36 | 0.96 | Amino acid |
| mannitol | 0.35 | 0.94 | Carbohydrate |
| 2-hydroxyhippurate (salicylurate) | 0.34 | 0.94 | Xenobiotics |
| 2-isopropylmalate | 0.34 | 0.94 | Carbohydrate |
| pyruvate | 0.34 | 0.76 | Carbohydrate |
| cortisone | 0.33 | 0.44 | Lipid |
| phosphate | 0.32 | 0.82 | Energy |
| hydroquinone sulfate | 0.32 | 0.92 | Xenobiotics |
| glucarate (saccharate) | 0.32 | 0.83 | Cofactors and vitamins |
| homocitrulline | 0.30 | 0.85 | Amino acid |
| 2,3-dihydroxyisovalerate | 0.30 | 0.97 | Cofactors and vitamins |
| N-acetyl-aspartyl-glutamate (NAAG) | 0.30 | 0.47 | Amino acid |
| quinolinate | 0.30 | 0.30 | Cofactors and vitamins |
| N1-methylguanosine | 0.26 | 0.97 | Nucleotide |
| 6-sialyl-N-acetyllactosamine | 0.26 | 0.69 | Carbohydrate |
| 4-vinylphenol sulfate | 0.25 | 0.91 | Xenobiotics |
| gentisate | 0.22 | 0.95 | Amino acid |
| urea | 0.22 | 0.49 | Amino acid |
| 1,6-anhydroglucose | 0.19 | 0.95 | Carbohydrate |
| succinate | 0.19 | 0.87 | Energy |
| tartarate | 0.18 | 0.81 | Xenobiotics |
| allantoin | 0.17 | 0.52 | Nucleotide |
| itaconate (methylenesuccinate) | 0.15 | 0.15 | Energy |
| pyroglutamylvaline | 0.15 | 0.91 | Peptide |
| 2-oxindole-3-acetate | 0.00 | 0.93 | Xenobiotics |
| xylose | 0.00 | 0.91 | Carbohydrate |
| phenol sulfate | 0.00 | 0.93 | Amino acid |
| glycylglycine | 0.00 | 0.38 | Peptide |
